# Optimization of cervical lymph node clinical target volume delineation in nasopharyngeal carcinoma: a single center experience and recommendation

**DOI:** 10.18632/oncotarget.23723

**Published:** 2018-06-05

**Authors:** Li Li, Yi Li, Jun Zhang, Qiuji Wu, Haijun Yu, Zheng Li, Conghua Xie, Yunfeng Zhou, Yahua Zhong

**Affiliations:** ^1^ Department of Radiation and Medical Oncology, Zhongnan Hospital of Wuhan University, Wuhan, Hubei, China; ^2^ Hubei Cancer Clinical Study Center, Wuhan University, Wuhan, Hubei, China

**Keywords:** nasopharyngeal carcinoma, intensity-modulated radiation therapy, cervical lymph node, clinical target volume

## Abstract

Nasopharyngeal carcinoma (NPC) are characterized by distinct lymph node metastasis patterns. In order to minimize cervical lymph node irradiation volume, 379 NPC patients with metastatic cervical lymph nodes were eligible for geographic mapping. All lymph nodes were mapped into simulation computed tomography images of a template lymph node negative patient. The proportions of retropharyngeal lymph nodes (RLNs), Level Ib, II, III, IV, Va, Vb and supraclavicular (SCV) lymph nodes were 6.9%, 0.5%, 55.25%, 20.4%, 8.2%, 4.9%, 3.1% and 0.75%, respectively. Based on their distribution profile, we proposed the following modifications: 1. the lateral border of RLNs clinical target volume (CTV) be the medial edge of the internal carotid artery above the level of mastoid process, the medial border be adjacent to the cervical vessels below the free edge of the soft palate; 2. the submandibular gland should not be included in Level Ib; 3. Level II should include the posterior belly of digastric muscle, and the space between the posterior edge of submandibular gland and the anterior edge of sternocleidomastoid muscle; 4. the anterior border of Level III and IV should gradually shift backwards and the CTV only include part of the cervical vessels below the level where the thyroid gland appears; 5. the space of the posterior edge of trapezius muscle also should be included if there are metastatic lymph nodes in the transverse cervical vessle plexus. Our recommendations might adequately encompass metastatic lymph nodes while sparing the organs at risk and reducing adverse events.

## INTRODUCTION

Nasopharyngeal carcinoma (NPC) is one of the most common malignant tumors in south China [1–4]. Radiotherapy is the predominant treatment modality [5]. With the development of modern radiotherapy equipments, treatment planning systems and the application of intensity-modulated radiation therapy (IMRT), the 5-year survival rate of NPC has increased from 50% in the 1970s to 80% in the 2000s [6]. Besides substantial efficacy improvement, IMRT also reduces some of the normal tissue complications such as mouth restriction [7], xerostomia [8, 9], radiation-induced brain injury [10, 11]. However, certain complications including acute radiation-induced mucositis [12], swallowing pain [13], radiation-induced skin injury [14] are still prominent, probably because a part of the cervical lymph node target volumes are larger. At present, the definition of the clinical target volume (CTV) of cervical lymph node in NPC is still in debate [14, 15]. The current delineation protocol is mainly based on DAHANCA, EORTC, GORTEC, RTOG consensus guidelines (hereafter referred to as Guidelines), which suggest CT-based delineation of lymph node volumes on the basis of lymph node anatomy and surgical findings from N0 patients [16, 17]. While the Guidelines are proposed for all head and neck cancers, NPCs are characterized by distinct lymph node metastasis patterns and merit a specific definition of lymph node CTVs [18, 19]. In order to specify the cervical lymph node irradiation volume to reduce the acute and long-term adverse effects, we first analysed the distribution profile of metastatic cervical lymph nodes in patients with NPC. Then we summarized differences between the consensus and the lymph node target volume delineation. We further proposed an optimized protocol of CTV delineation of cervical lymph nodes in NPC.

## RESULTS

### Case characteristics

379 cases of NPC were eligible for analysis, including 287 males and 92 females. The median age was 49 (15–82) years old. A total of 5949 metastatic lymph nodes were identified and mapped. The proportions of stage I, II, III, and IVa diseases (according to the American Joint Committee on Cancer (AJCC) 2009 cancer staging system [20]) were 1.36%, 5.46%, 53.58% and 39.59% respectively. The proportions of N1, N2 and N3 lymph nodes were 6%, 74%, 20%, respectively.

### Lymph node metastasis probabilities

The proportions of retropharyngeal lymph nodes (RLNs), Level Ib, II, III, IV, Va, Vb and supraclavicular (SCV) lymph nodes were 6.9%, 0.5%, 55.25%, 20.4%, 8.2%, 4.9%, 3.1% and 0.75%, respectively.

### Distribution patterns of cervical lymph nodes in each level and suggestions of the CTV delineation

**Figure 1:**
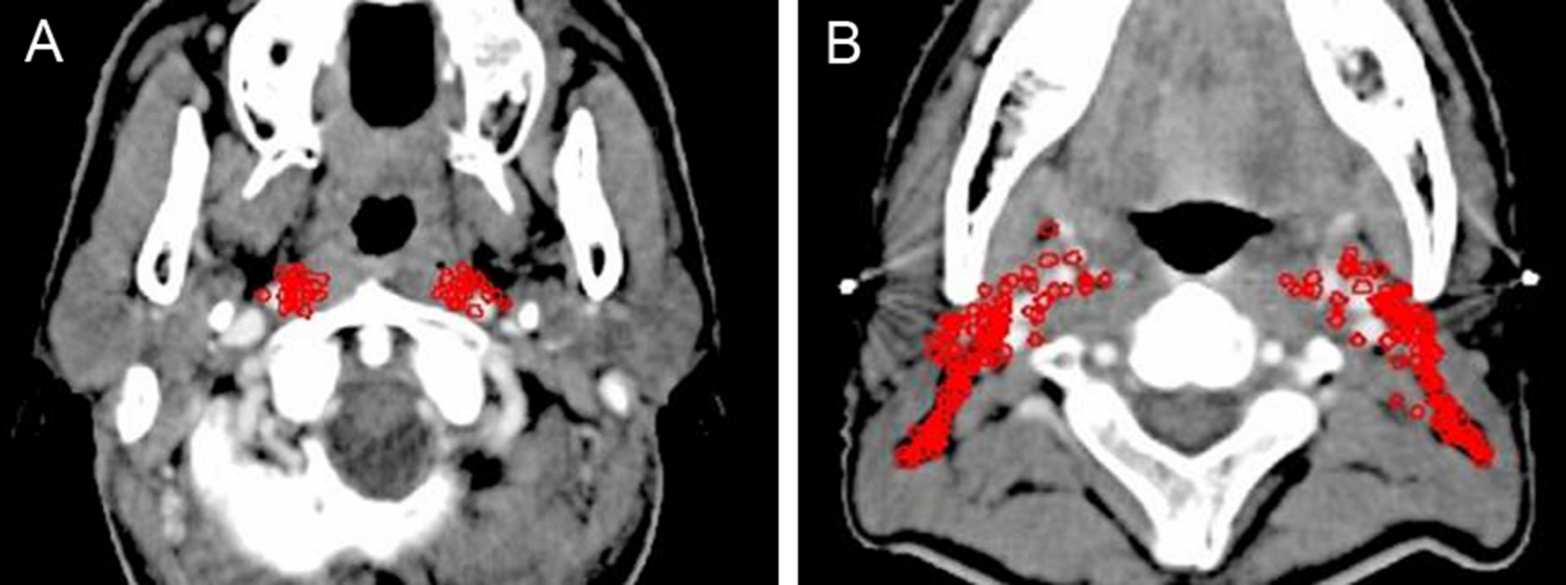
Lymph node distribution pattern of retropharyngeal lymph nodes (**A**) Lymph nodes distribution above the level of the mastoid process. (**B**) Lymph nodes distribution below the free margin of the soft palate. Images are representative axial planes of the lymph node level.

**Figure 2:**
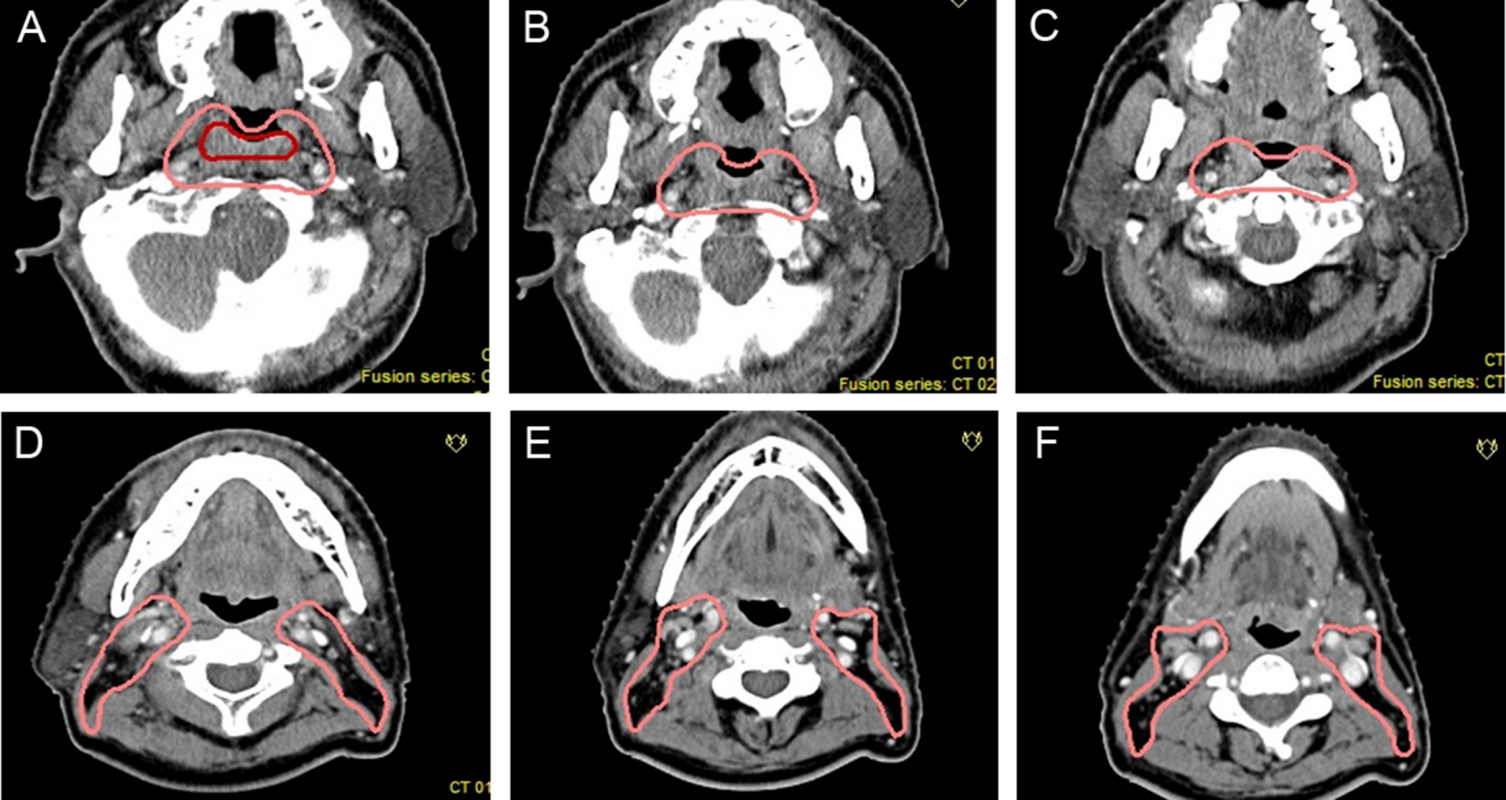
Proposed CTVdelineation protocol for retropharyngeal lymph nodes (**A**–**C**) The lateral border should be set at the medial edge of the internal jugular vein above the mastoid process. (**D**–**F**) The medial edge should be close to the cervical vascular below the free edge of the soft palate. The red circles indicates GTV of the primary lesion and pink circles indicate CTVs of RLNs and primary lesions.

2.3.1 RLNs: Above the level of the mastoid process, the RLNs extended to the medial edge of the internal carotid artery or its surrounding, but did not go beyond the medial edge of the internal jugular vein (Figure 1A). Below the free margin of the soft palate, the RLNs were adjacent to the medial edge of the internal carotid artery. There were no metastatic lymph nodes of the median group (Figure 1B). The RLNs are often included in one integrated CTV with the nasopharyngeal primary lesion or adjacent cervical lymph nodes (Level II). Therefore, we suggest that the lateral border of the CTV be the medial edge of the internal jugular vein above the mastoid process (Figure 2A–2C), the medial edge be close to the cervical vascular below the free edge of the soft palate (Figure 2D–2F).

**Figure 3:**
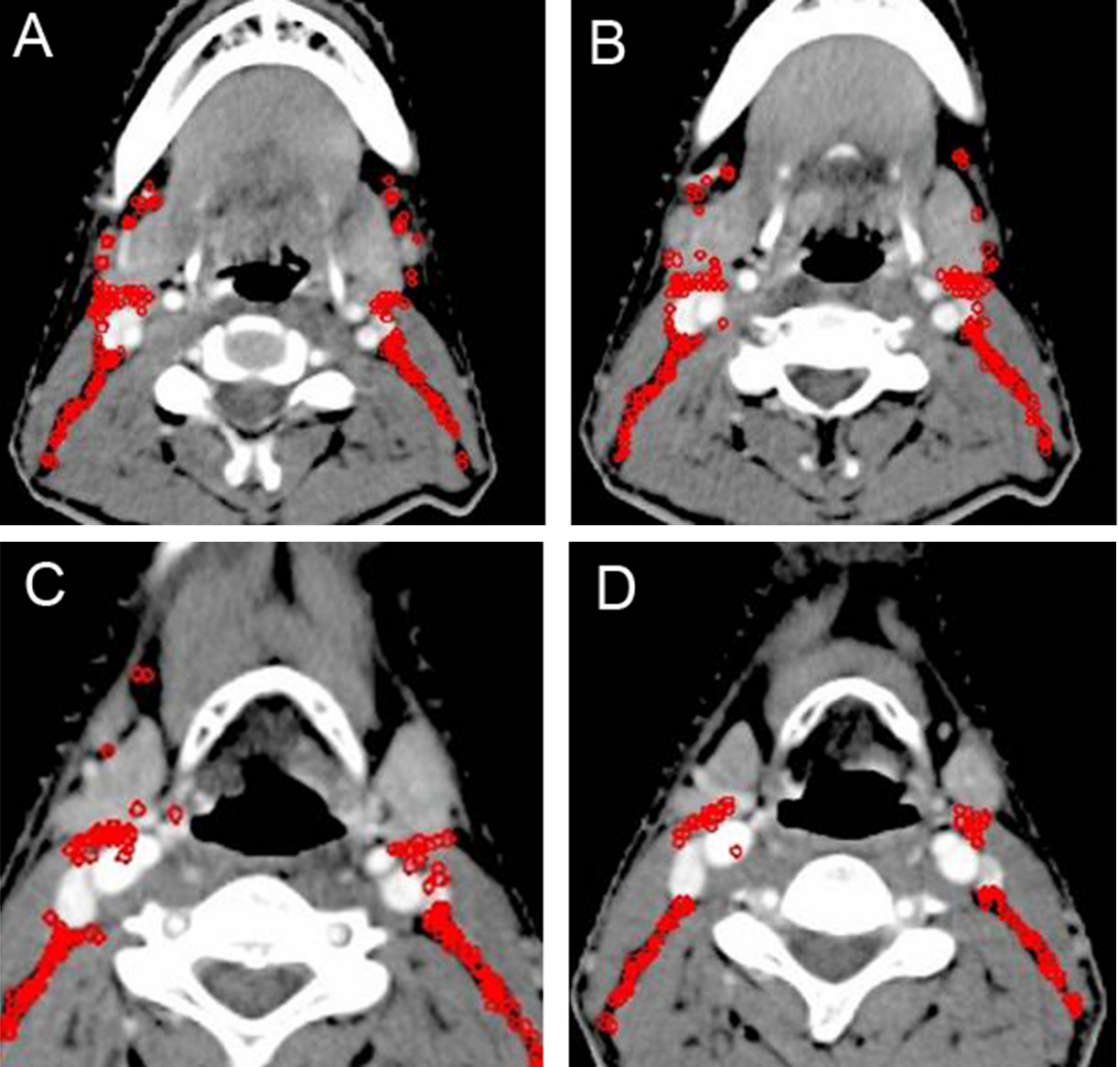
Lymph node distribution patterns of Level Ib lymph nodes (**A**–**C**) The lymph nodes were distributed in the anterolateral space of submandibular glands. (**D**) There was no metastatic lymph node below the anterior belly of digastric muscle. Images are representative axial planes of the lymph node level.

**Figure 4:**
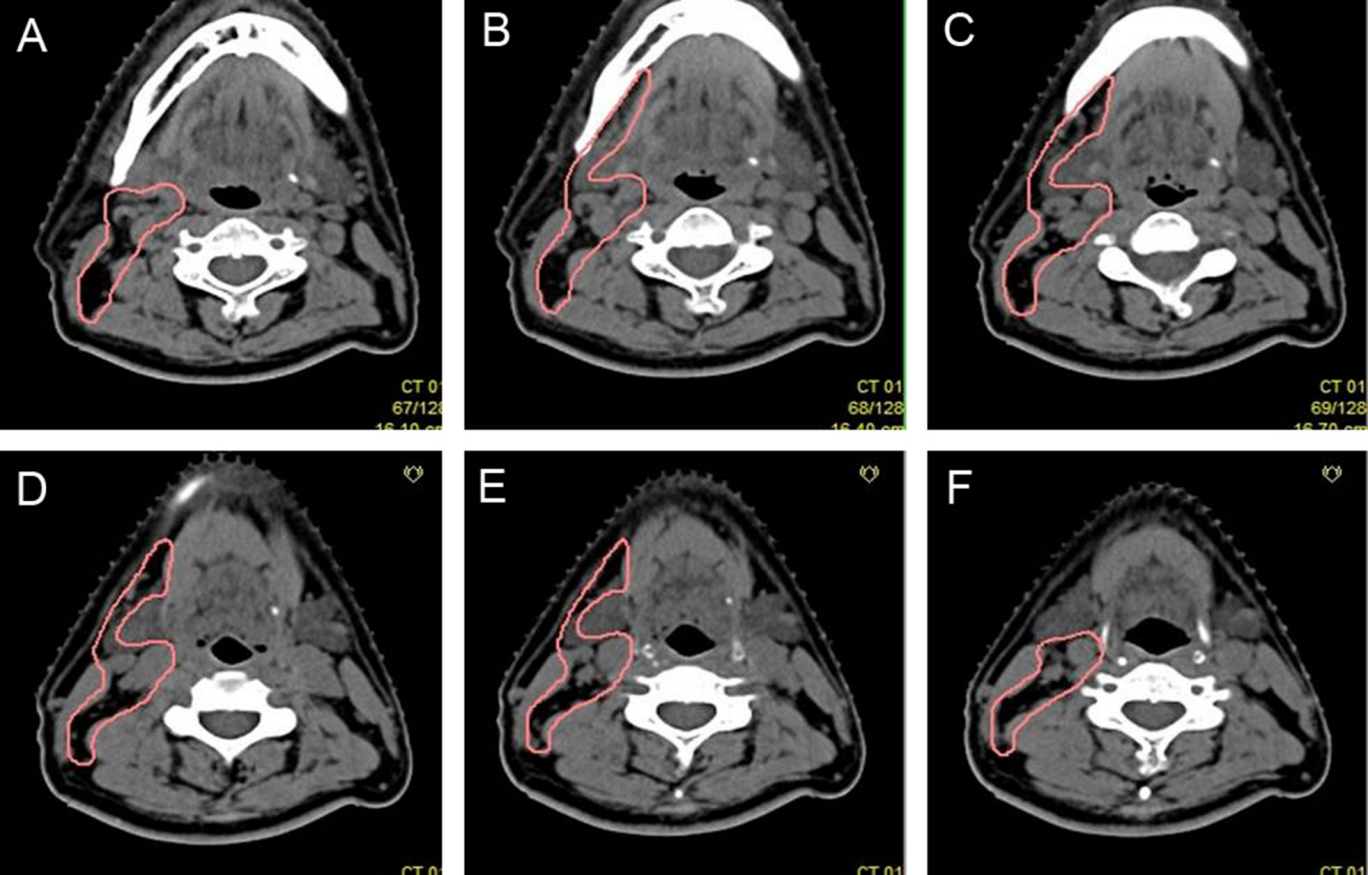
Proposed CTV delineation protocol for Level Ib lymph nodes (**A**–**E**) The anterolateral space of submandibular gland should be included in Level Ib. (**F**) The inferior border is the level where the anterior belly of digastric muscle disappears.

2.3.2 Level Ib: The cervical lymph nodes of Level Ib were distributed in the anterolateral space of submandibular glands. There was no metastatic lymph node inside the submandibular gland (Figure 3A–3C). There was no metastatic lymph node below the anterior belly of digastric muscle in Level Ib (Figure 3D). So we suggest that the CTV should include the anterolateral space of submandibular glands, but the submandibular gland itself should not be included. The inferior border of Level Ib is defined as the level where the anterior belly of the digastric muscle disappears (Figure 4A–4F).

**Figure 5:**
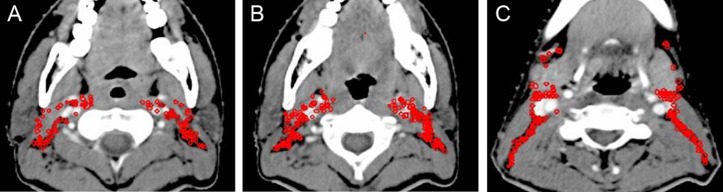
Lymph node distribution patterns of Level II lymph nodes (**A**–**C**) The lymph nodes were distributed around the digastric muscle, carotid sheath and extended to the space between the sternocleidomastoid muscle and paraspinal muscles. Images are representative axial planes of the lymph node level.

**Figure 6:**
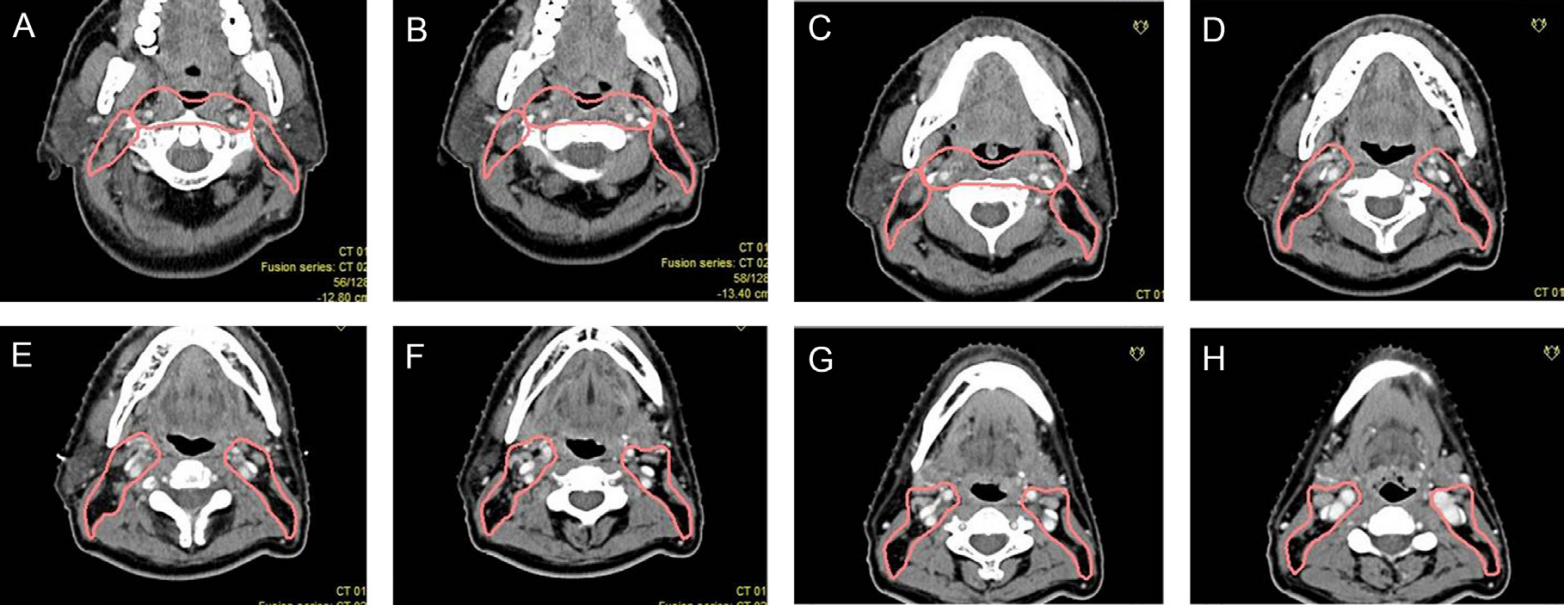
Proposed CTV delineation protocol for Level II lymph nodes (**A**–**D**) The CTV include the posterior belly of digastric muscle. (**E**–**H**) The space between the posterior edge of submandibular gland and the anterior edge of sternocleidomastoid muscle should be included.

2.3.3 Level II: The lymph nodes of Level II were distributed around the digastric muscle, carotid sheath and extented posteriorly to the space between the sternocleidomastoid muscle and paraspinal muscles. The posterior space of the submandibular gland and the anterior space of the sternocleidomastoid muscle were also predilection sites of lymph nodes metastasis (Figure 5A–5C). So we propose that the CTV of Level II should include the posterior belly of digastric muscle (Figure 6A–6D), carotid sheath, the space between the sternocleidomastoid muscle and paraspinal muscles, the posterior space of the submandibular gland, and the anterior space of the sternocleidomastoid muscle (Figure 6E–6H).

**Figure 7:**
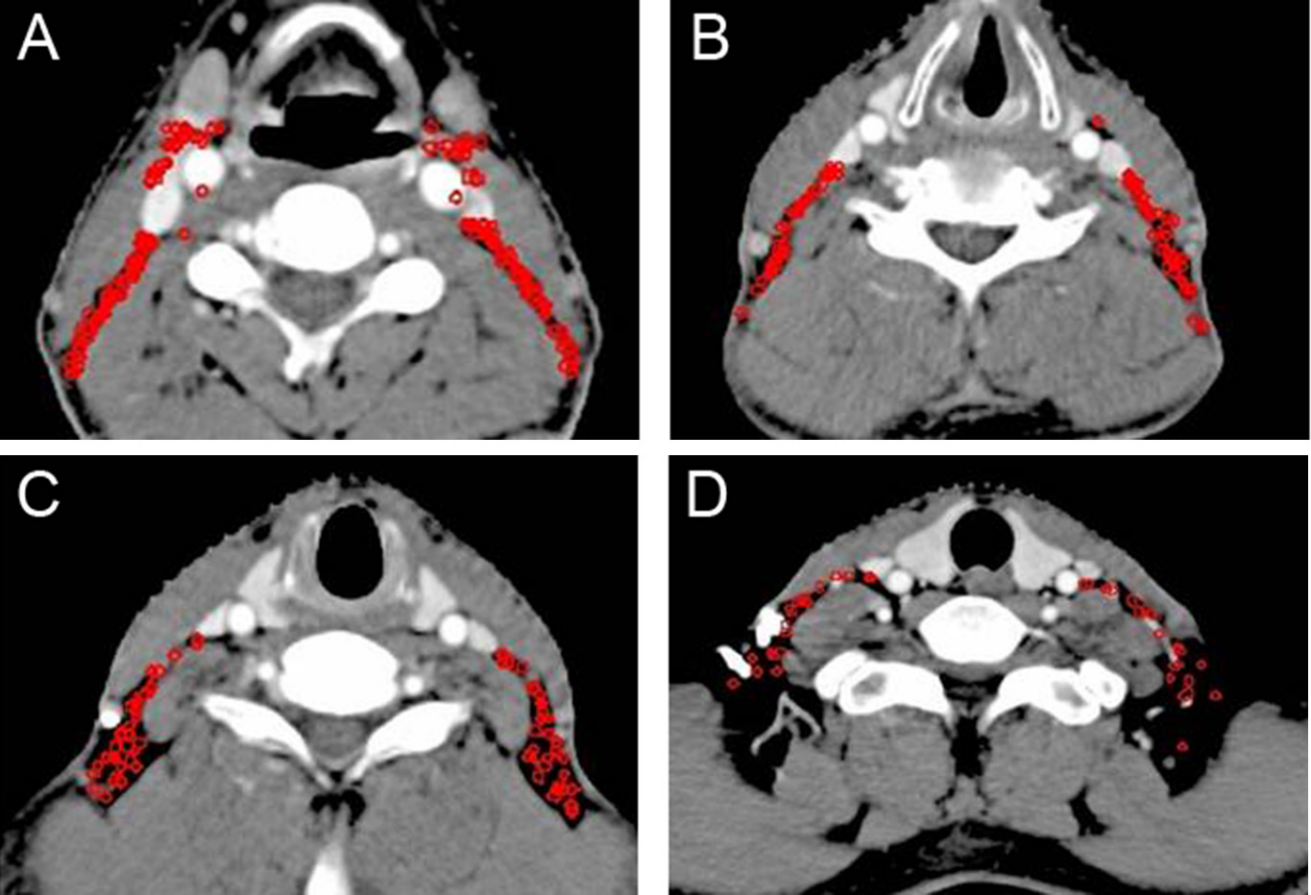
Lymph node distribution patterns of Level III and IV lymph nodes (**A**–**D**) The lymph nodes were mainly distributed behind the posterior edge of the carotid artery and internal jugular vein. Images are representative axial planes of each lymph node level.

**Figure 8:**
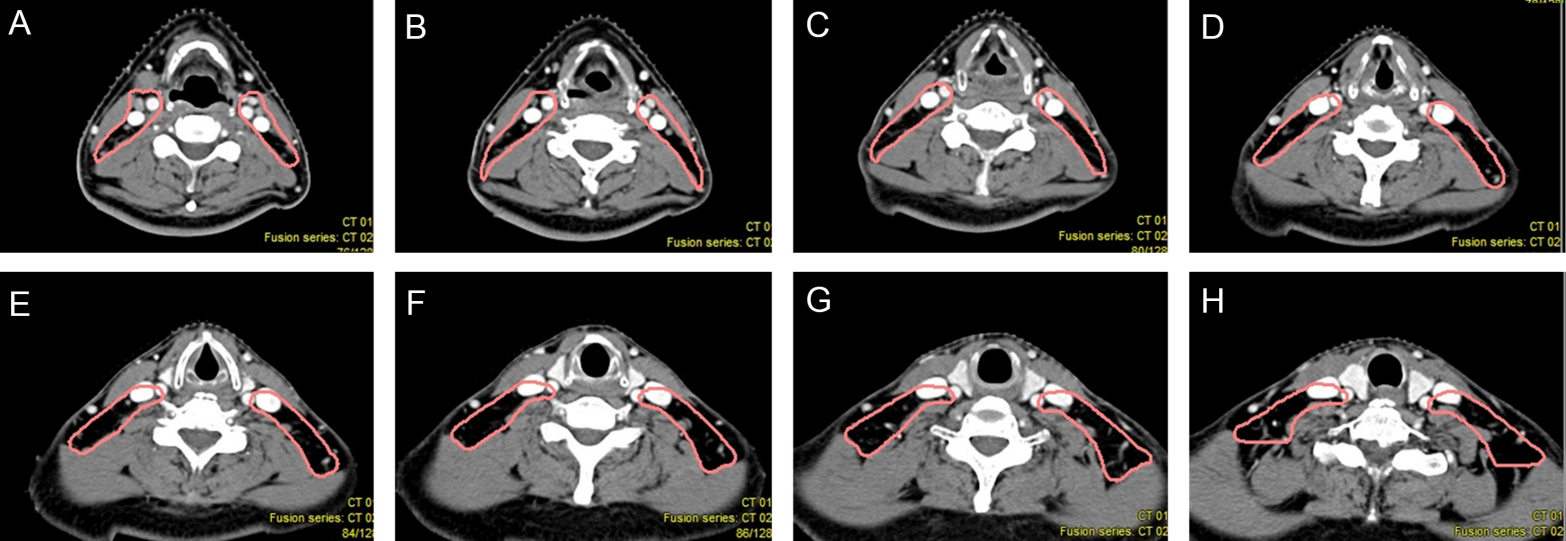
Proposed CTV delineation protocol for Level III and IV lymph nodes (**A**–**D**) The anterior boarder should gradually shift backwards. (**E**–**H**) The CTV should only include part of the cervical vessels below the level where the thyroid gland appears.

2.3.4 Level III and IV: Below the level of the thyroid gland, the number of metastatic lymph nodes in the space anterior of blood vessles declined gradually. Most of them were distributed behind the posterior edge of the carotid artery and the internal jugular vein (Figure 7A–7D). As the level descended, the anterior space of vascular narrowed down, so we suggest that the anterior boarder of the CTV should gradually shift backwards (Figure 8A–8D) and the CTV only include part of the cervical vessels below the level where the thyroid gland appears (Figure 8E–8H).

**Figure 9:**
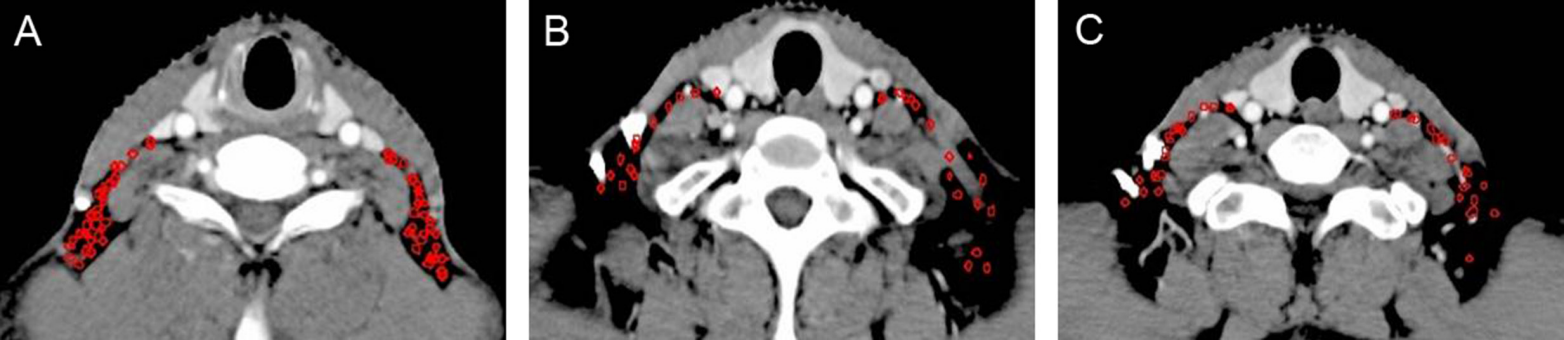
Lymph node distribution patterns of Level V lymph nodes (**A**–**C**) The lymph nodes were mainly distributed in the transverse cervical vessle plexus, and also in the space behind the anterior border of trapezius muscle. Images are representative axial planes of the lymph node level.

**Figure 10:**
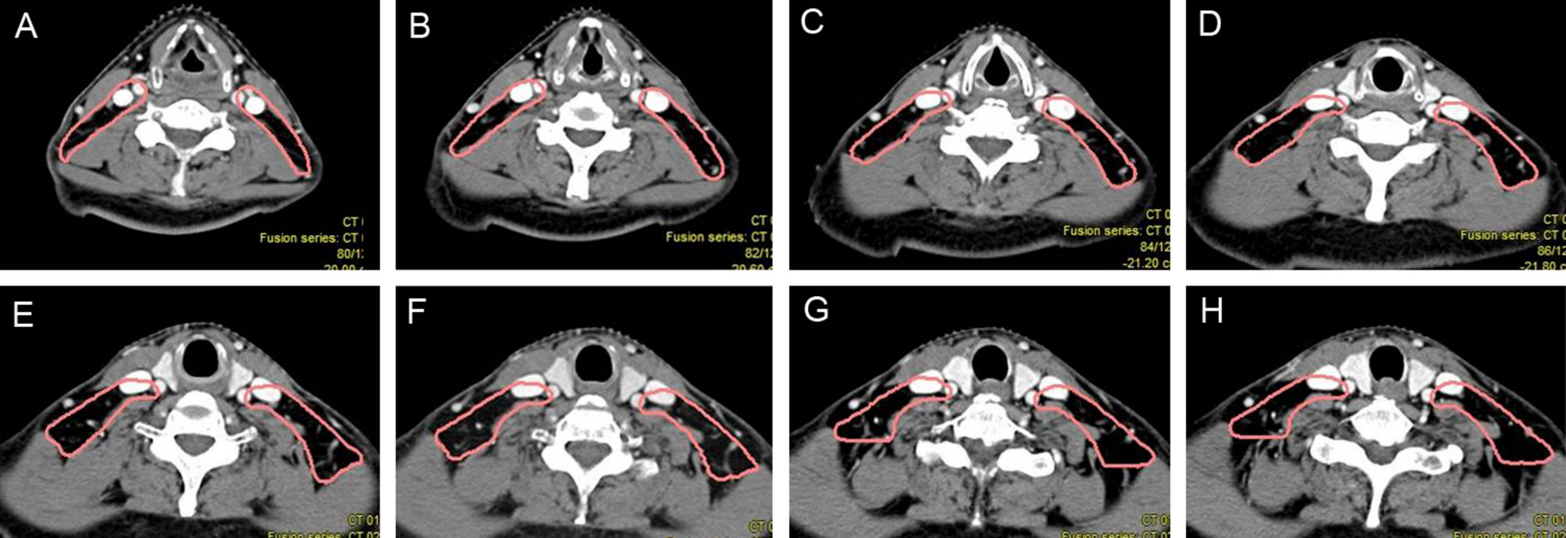
Proposed CTV delineation protocol for Level V lymph nodes (**A**–**H**) the CTV should include the space between the posterior edge of sternocleidomastoid muscle and the anterior edge of trapezius muscle.

2.3.5 Level V: At level V, metastatic lymph nodes were mainly distributed in the transverse cervical vessle plexus. We could also detect metastatic lymph nodes in the space behind the anterior border of trapezius muscle. However, there was no lymph node around the external jugular vein. Metastatic lymph nodes could also been seen in the posterior edge of the trapezius muscle at the level of the transverse cervical vessle plexus (Figure 9A–9C). So we suggest that the CTV should include the space between the posterior edge of sternocleidomastoid muscle and the anterior edge of trapezius muscle. The space of behind the anterior edge of the trapezius muscle should also be included if there are metastatic lymph nodes intransverse cervical vessle plexus. The external jugular vein should not be included in the CTV (Figure 10A–10H).

**Figure 11:**
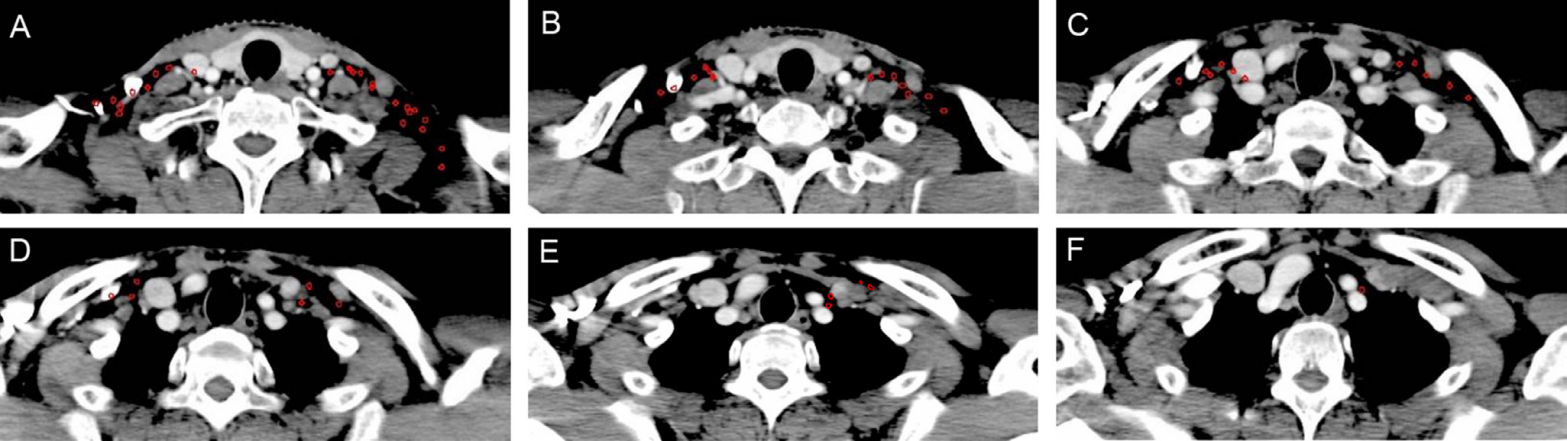
Lymph node distribution patterns of supraclavicular lymph nodes (**A–F**) Images are representative axial planes of the lymph node level.

**Figure 12:**
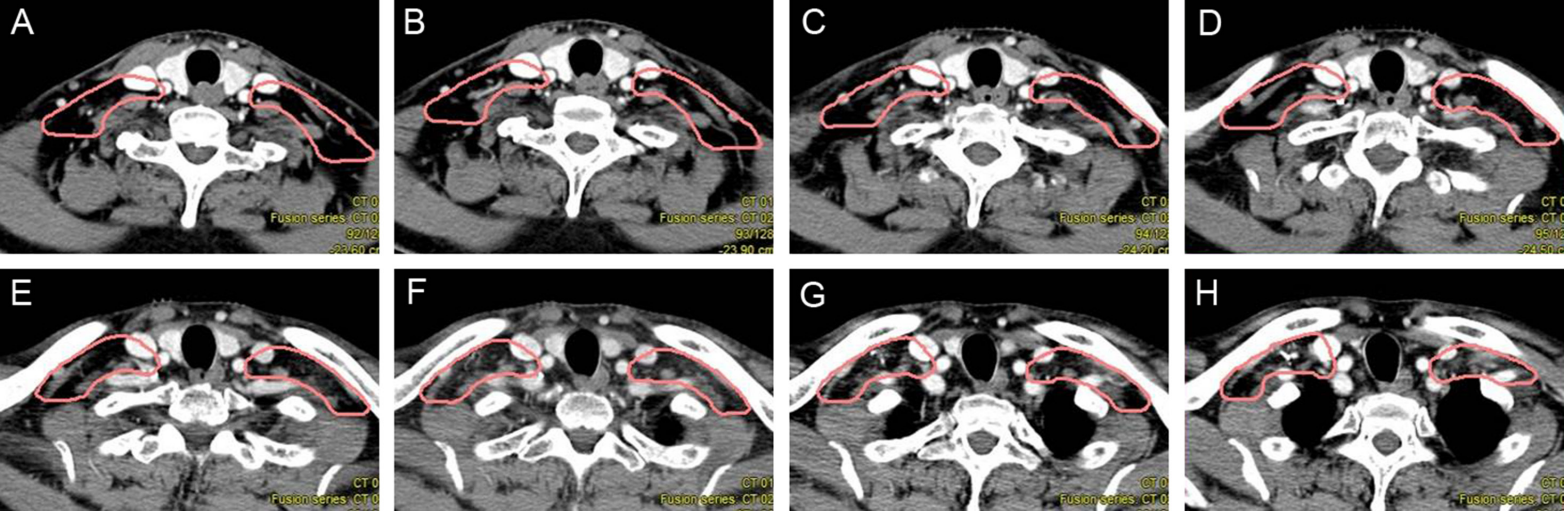
Proposed CTV delineation protocol for supraclavicular lymph nodes (**A–H**) Images are representative axial planes of the lymph node level.

2.3.6 Supraclavicular (SCV) lymph nodes: Metastatic lymph nodes were distributed in the space that was confined medially by the lateral edge of the common carotid artery and the jugular vein, laterally by the internal edge of sternocleidomastoid muscle and the clavicle, posteriorly by the anterior edge of the posterior scalene, and inferiorly by the superior edge of the subclavian vein (Figure 11A–11F). Therefore, we propose that the medial border of the CTV should include part of the common carotid artery and jugular vein. The lateral border should be the sternocleidomastoid muscle and the clavicle. The posterior border should be the anterior edge of the posterior scalene. The inferior border should be the superior edge of the subclavian vein (Figure 12A–12H).

## DISCUSSION

Lymph node metastasis is of common occurence in NPC at early stage [21]. 36%–45% patients of NPC presented with the initial symptoms of neck lymphadenectasis, 60%-90% of them presents with cervical lymph node metastasis when newly diagnosed [22]. There are certain disciplines in the distribution of lymph node metastasis [19, 23]. Radical irradiation of the nasopharyngeal primary lesion along with prophylactic cervical node irridation has long been the standard care for NPC patients with considerable risk of relapse [22]. There have been a number of studies about lymphatic metastasis, most of which only focus on the distribution probabilities in each district [21, 24, 25]. An innovative study of a Fudan University team using a similar method as ours in 259 NPC patients showed that 218 patients (84.2%) were presented with metastatic lymph nodes, of whom 65 patients (29.8%) with metastatic lymph node in the left neck, 60 (27.5%) in the right neck and 93 (42.7%) patients in both necks. The probility of skip metastasis is 2.3% [26]. All patients in our study were presented with cervical lymph node metastasis at diagnosis, with a high probability of lymph node metastasis in ipsilateral and contralateral Levels II and RLNs. The probability is gradually decreased in Levels III, IV, V and Ib. The probability of Level Ia and VI is 0%. The rule of lymph node metastasis is associated with lymphatic drainage of the nasopharynx, which is rich and crossing [27]. The lymphatic vessels from the lateral and the posterior wall of nasopharynx finally drain into the lymph nodes of the internal jugular vein. The RLNs, deep lymph nodes below the mastoid process, internal jugular vein-digastric muscle lymph nodes and the internal jugular vein lymph nodes are station 1 lymph nodes of NPC [28]. Only when these deep cervical lymph nodes cause extensive lymphatic blockage that the retrograde to the submandibular, submental, occipital, auricular and other lymph nodes could be seen.

Different CTV delineation strategies for cervical lymph nodes have been proposed based on studies on imagings and cadaver dissections, of which the DAHANCA, EORTC, GORTEC, RTOG consensus guidelines are more accepted ones. As an improvement to the proposition that the lateral border of the RLNs be at the medial edge of internal carotid artery by Guidelines [16], we found that lymph nodes were mainly distributed in the internal carotid artery and its surroundings above the level of mastoid process. So we suggested that the lateral border of RLNs be the medial edge of the internal jugular vein above the level of mastoid process, which would reduce the risk of missing metastatic lymph nodes in the lateral edge of the internal carotid artery. Below the free edge of the soft palate, we found that the metastatic lymph nodes were adjacent to the medial edge of the internal carotid artery, and there was no intermediate group RLNs metastasis. Therefore, we proposed that the medial border be adjacent to the neck vessels below the free edge of the soft palate, which is different from the recommendation by the Guidelines that set the medial border to the midline [16]. This can reduce the irradiation dose of the posterior edge of oropharynx below the soft palate, and decrease the risk of acute radiation-induced mucositis [29] and late dysphagia [30].

In a pioneer research, 3-dimensional conformal radiation therapy (3DCRT) and IMRT were shown to achieve better conformity, dose homogeneity, and organ preservation compared with 2D techniques in NPC radiation treatment [31]. Compared with 2DRT, IMRT reduced the radiation-induced toxicities [32]. The trials from Pow et al. and Kam et al. indicated that IMRT for patients with NPC could preserve parotid function and improve corresponding subscale scores on quality of life [8, 33]. The most common radiation-related complication was xerostomia. A retrospective analysis from Taiwan showed that a total of 745 patients had xerostomia among 849 NPC patients, and the 5-year complication-free (CF) rate was 9.7%. Among them, 310 patients developed xerostomia during the course of radiotherapy [7]. Guidelines [16] recommend that the CTV of Level Ib should include the submandibular gland, However, we found that lymph nodes of Level Ib were mainly distributed in the anterolateral space of submandibular gland. There was no metastatic lymph node in submandibular glands. Therefore, we suggested that the CTV should include the anterolateral space of submandibular gland, but the submandibular gland itself should be spared. This strategy would reduce the incidence and the severity of xerostomia. In addition, Guidelines recommended that the inferior border of Level Ib be the inferior edge of hyoid bone. However, we found no metastatic lymph node below the anterior belly of digastric muscle in Level Ib. Therefore, we suggested that the inferior border of Level Ib should be defined as the level where the anterior belly of digastric muscle disappeared. By doing this, we chould effectively reduce the incidence of submandibular subcutaneous edema.

In the Guidelines recommondation [16], the posterior belly of digastric muscle was not included in Level II. However, we found that there were metastatic lymph nodes in the lateral and posterior part of the digastric muscle, which might be omitted otherwise if we delineate the target volume according to Guidelines. Therefore, it was suggested that the CTV of Level II should include the posterior belly of digastric muscle. We also found that the anterior space of the sternocleidomastoid muscle and the posterior space of the submandibular gland is a predilection site for lymph node metastasis. Therefore, these spaces were also recommended to be included in the CTV of Level II. As the levels descend, the number of metastatic lymph nodes of the anterior space of blood vessles declined gradually. So we suggested that the anterior space of the CTV should gradually shift backwards as the anterior space of the descending vascularization narrowed down. Since there was metastasitic lymph node in the posterior edge of the submandibular gland, the CTV should include part of the anterior vascular space and the carotid artery and the jugular vein until the thyroid gland appears. The lymph nodes were distributed in the posterior border of the carotid artery and the jugular vein below the level of the thyroid gland. Therefore, below the level of the thyroid gland, we suggested that the CTV only include part of the carotid artery and the jugular vein, which could protect the thyroid gland and the hypopharynx. The metastatic lymph nodes were mainly distributed in the transverse cervical vessle plexus. But we could also detect metastatic lymph nodes behind the anterior border of the trapezius muscle. If there are metastasitic lymph nodes in the transverse cervical vessle plexus, the CTV should be appropriately expanded. We further suggested that the external jugular vein should not be included. This is because the external jugular vein mainly collect venous blood of the auricle, occipital and superficial layer of the anterior aspect of the neck, but do not collect lymphatic drainage of the nasopharynx. This would reduce the risk of subcutaneous edema caused by external jugular vein irradiation. Notably, as indicated by the Guidelines, this study only applies to the patients with negative or scarce, small cervical lymph node that require prophylactic irradiation. In case of positive and multiple lymph nodes, the CTV should be expanded accordingly to ensure adequate safety margin.

Radiotherapy is the primary treatment for NPC patients. In recent years, we have seen the wide application of IMRT, which improves the coverage of target volumes of the primary nasopharyngeal tumors and the involving lymph nodes. IMRT also offers appropriate protection to the spinal cord, the brain stem and salivary glands that considerably improved the quality of life of NPC patients. In this study, we analysed the distribution profiles of the cervical lymph node metastasis of NPC, and we further put forward our recommendations on the CTV outline of the cervical lymph nodes. This study emphasized that our recommendations might help to reduce the acute complications and late sequelaeas compared to Guidelines recommondations. The long-term follow-up are still underway and we will further report our analysis on acute and chronic side effects with the usage of this optimized protocol for lymph node delineation in NPC patients.

## MATERIALS AND METHODS

### Clinical data

From January 2012 to June 2016, a total of 379 histologically diagnosed lymph node positive and nondistal-metastatic NPC patients were treated primarily with IMRT according to an institutional review boarder approved treatment protocol at Zhongnan Hospital of Wuhan University.

4.2 Computed tomography (CT) scans and target volume delineation strategies: All patients were immobilized in the supine position with a thermoplastic mask. CT with intravenous contrast using a 3-mm slice from the head to the level of 2 cm below the sternoclavicular joint was performed for treatment planning. CT images were used for target volume delineation, and planning CT with Magnetic resonance imaging (MRI)/CT fusion using a co-registration software (Oncentra MasterPlan^®^version1.5, Nucletron B.V., Veenendaal, the Netherlands) was performed for all patients.

4.3 CT template preparation and lymph node mapping: An N0 NPC patient was selected. The CT images of this patient were acquired as indicated in 2.2. Set these enhanced CT images of NPC as template images, and the blood vessels, muscles, and bony landmarks as location references for lymph nodes. Metastatic cervical lymph nodes of 379 NPC patients were identified and mapped into the template CT images, and the distribution patterns of metastatic cervical lymph nodes in NPC were analysed [34].

### Diagnostic criteria of lymph node metastasis

refer to Michiel and Lam cervical lymph node metastasis of the imaging diagnostic criteria [18, 35, 36]: ①cross-sectional minimum diameter ≥ 10 mm; ②central necrosis or ring enhancement; ③three or more lymph nodes were clustered in the same region, and with a minimum diameter of ≥ 8 mm; ④RLNs: cross-sectional minimum diameter ≥ 4 mm.
